# The Potential of Fluocinolone Acetonide to Mitigate Inflammation and Lipid Accumulation in 2D and 3D Foam Cell Cultures

**DOI:** 10.1155/2018/3739251

**Published:** 2018-11-22

**Authors:** Luong T. H. Nguyen, Aristo Muktabar, Jinkai Tang, Yee Shan Wong, Colby S. Thaxton, Subbu S. Venkatraman, Kee Woei Ng

**Affiliations:** ^1^Nanyang Technological University, School of Materials Science and Engineering, 50 Nanyang Avenue, Singapore 639798, Singapore; ^2^Northwestern University, Feinberg School of Medicine, Department of Urology, Chicago, IL 60611, USA

## Abstract

Inflammation plays an important role in all stages of atherosclerosis development. Therefore, the use of anti-inflammatory drugs could reduce the risk of major adverse cardiovascular events due to atherosclerosis. Herein, we explored the capacity of fluocinolone acetonide (FA), a glucocorticoid (GC), in modulating foam cell formation and response. Human THP-1 derived foam cells were produced using 100 *μ*g/mL oxidized low-density lipoproteins (OxLDL) and fetal bovine serum (1 and 10%). 2D cultures of these cells were treated with FA (0.1, 1, 10, and 50 *μ*g/mL) in comparison with dexamethasone (Dex). Results showed that treatment with 0.1 and 1 *μ*g/mL FA and Dex improved foam cell survival. FA and Dex also inhibited inflammatory cytokine (CD14, M-CSF, MIP-3*α*, and TNF-*α*) secretion. Notably, at the concentration of 1 *μ*g/mL, both FA and Dex reduced cholesteryl ester accumulation. Compared to Dex, FA was significantly better in reducing lipid accumulation at the therapeutic concentrations of 1 and 10 *μ*g/mL. In a novel 3D foam cell spheroid model, FA was shown to be more effective than Dex in diminishing lipid accumulation, at the concentration of 0.1 *μ*g/mL. Taken together, FA was demonstrated to be effective in preventing both lipid accumulation and inflammation in foam cells.

## 1. Introduction

Atherosclerosis is a chronic artery inflammatory disease, which can lead to fatal acute myocardial infarctions, sudden coronary deaths, and ischemic stroke [1, 2]. Cardiovascular risk factors such as chronic smoking and hypertension can cause the dysfunction of vascular endothelium, increasing the permeation of cholesterol-containing low-density lipoproteins (LDL) [3]. These LDL particles are susceptible to various modifications including oxidation, enzymatic and nonenzymatic cleavage, and aggregation, leading to the recruitment of monocytes and T cells to the arterial wall [1, 4]. Thereafter, monocytes differentiate into tissue macrophages which ingest the accumulated normal and modified lipoproteins and transform into foam cells, a term that reflects the microscopic appearance of these lipid-laden macrophages [5]. Although macrophage clearance of lipoproteins is beneficial at the beginning of this immune response, negative feedback following the uptake of lipoproteins makes these cells become excessively engorged with lipids. The resultant lipid metabolism dysregulation changes the macrophage phenotype and compromises critical immune functions [1].

Due to the significant role that inflammatory processes play in the progression of atherosclerosis, several anti-inflammatory drugs that aim to reduce the risk of major adverse cardiovascular events are now being investigated [4, 6]. GCs are among the most widely used anti-inflammatory drugs [7]. GCs can easily diffuse through cellular membranes and bind to glucocorticoid receptors (GRs) in the cytoplasm. The binding of GCs to GRs will cause the dissociation of molecular chaperones which exposes nuclear localization signals on GRs, resulting in the rapid translocation of the GC-GR complexes into cellular nuclei [7]. There are three possible mechanisms that GCs can act within the cells: (1) at high doses of GCs, the GC-GR complex binds as a homodimer to glucocorticoid response elements (GREs), which typically leads to an increased transcription of anti-inflammatory genes; (2) under inflammatory stimuli, a dimer of p50 and p65 nuclear factor kappa B (NF-*κ*B) proteins translocates to the nucleus and binds to NF-*κ*B elements, which stimulates the transcription of multiple inflammatory proteins (cytokines, chemokines, cell adhesion molecules, complement factors, and receptors for these molecules). At lower levels of GCs, the GC-GR complex physically interacts with NF-*κ*B in the nucleus to block its transcriptional activity; and (3) GCs can also have rapid effects on inflammation* via* nongenomic mechanisms by inhibiting the proteins that stabilize the mRNA of inflammatory genes, leading to a reduction of inflammatory protein expression [7, 8]. In addition to these classical cytosolic GRs, GCs have recently been found to exert rapid nongenomic effects, within seconds of exposure, by the activation of membrane bound GRs [9].

Dexamethasone (Dex) is the most common GC used in atherosclerotic treatment. It was shown that Dex at 1 mg/kg daily for 7 days after balloon arterial injury resulted in reduced macrophage accumulation by 96% and 77%, respectively, in the tunica intima and tunica media of arteries in cholesterol fed rabbits [10]. Dex-loaded liposomes also significantly inhibited monocyte and macrophage migration* in vitro* and diminished proinflammatory cytokine secretion (specifically tumor necrosis factor (TNF), interleukin-1 beta (IL-1*β*), and IL-6) under inflammatory conditions [11]. Additionally, a significant reduction of 49% in vein graft thickening at 28 days was observed after a short-term 7-day Dex treatment in hypercholesterolemic ApoE3-Leiden transgenic mice, which was possibly due to the reduced early expression of proinflammatory cytokines [12]. Not only did it affect inflammatory process, but also Dex was found to lower cholesterol accumulation. Tauchi et al. demonstrated that dexamethasone palmitate- (DP-) LDL complex inhibited murine foam cell formation* in vitro* [13] and considerably decreased cholesteryl ester (CE) accumulation in the aorta of atherogenic mice [14]. Similarly, Dex-incorporated liposomes were shown to obstruct cellular CE accumulation* in vitro *[15] and induced excellent antiatherosclerotic effects by lowering aortic CE levels in atherogenic mice [16].

Fluocinolone acetonide (FA) is a GC primarily used to reduce skin inflammation and relieve itching [17], as well as treat mucosal disorders [18] and endodontic conditions by achieving anti-inflammatory effects by inhibiting the NF-*κ*B pathway [19]. However, FA has not been explored for treating atherosclerosis or other cardiovascular diseases. In this study, we aimed to evaluate the potential of FA in atherosclerosis treatment by investigating its effects on the proliferation, lipid accumulation, inflammation, and cytokine expression of human THP-1 derived foam cells, in comparison to Dex.

## 2. Materials and Methods

### 2.1. Foam Cell Formation

Human monocytic THP-1 cells (ATCC® TIB202™, USA) were cultured in Roswell Park Memorial Institute (RPMI) 1640 Medium (ATCC modification; Life Technologies, USA) containing 0.05 mM 2-mercaptoethanol (Sigma Aldrich, USA), 1% Penicillin-Streptomycin Solution (Pan Biotech, Germany), and 10% fetal bovine serum (FBS, Pan Biotech, Germany). The medium was replaced every 2–3 days by centrifuging these suspended cells at 125 g for 7 min. Cultures were maintained in a humidified incubator at 37°C with 5% CO_2_ and subcultured once cell concentration reaches 8 × 10^5^ cells/mL. THP-1 cells at passages 6–8 were used for all experiments. The monocytes were seeded at a density of 2 × 10^5^ cells/well in 24-well tissue culture plates (TPP, Switzerland) in a differentiation medium, growth medium supplemented with 50 ng/mL phorbol 12-myristate 13-acetate (PMA, Sigma Aldrich, USA), for 3 days to obtain macrophages. This differentiation medium contained either 1% or 10% FBS depending on experiments as described in the following sections and are referred to as DM-01 and DM-10, respectively. Subsequently, the macrophages were fed with 100 *μ*g/mL oxidized low-density lipoprotein (OxLDL, Alfa Aesar, USA) in the differentiation medium for 2 days, to produce foam cells.

### 2.2. Effects of FBS and OxLDL on Foam Cell Formation

To understand the role of FBS and OxLDL in foam cell formation, the THP-1 monocytes were suspended in DM-01 or DM-10 for 3 days. The resulting macrophages were then refreshed with the differentiation medium containing the same FBS concentration in the presence or absence of 100 *μ*g/mL OxLDL, for 2 days. The formation of foam cells was subsequently confirmed using Oil Red O (ORO) staining and OxLDL immunofluorescence staining.

### 2.3. Effects of FA and Dex on Foam Cell Behaviour

The THP-1 cells were differentiated in DM-10 for 3 days before treatment with DM-10 containing 100 *μ*g/mL OxLDL and glucocorticoids (FA (Cat. 1082381, International Laboratory, USA) or Dex (Cat. D4902, Sigma Aldrich, USA)) for 2 days. The concentrations of FA/Dex applied in this study were 0.1, 1, 10, and 50 *μ*g/mL. Cells cultured in the absence of FA/Dex were used as negative controls. The effects of FA/Dex on cell proliferation and lipid accumulation were evaluated using the PicoGreen assay, cholesterol quantification assay, and a cytokine array.

### 2.4. Foam Cell Spheroid Formation

A three-dimensional (3D) foam cell spheroid model was developed using the hanging droplet culture method to further evaluate the efficacy of FA and Dex. After foam cells were formed in DM-10 in a T-75 flask as described above, trypsin (Gibco, USA) was used to detach the cells. The foam cells were then centrifuged at 250 g for 5 min and resuspended to 1.25 × 10^6^ cells/mL. Subsequently, the cells were seeded into Perfecta3D® 96-well hanging drop plate (3D Biomatrix™, USA) and incubated for 4 days at 37°C with 5% CO_2_. Spheroids formed in this plate were harvested into Corning® Costar® Ultra-Low Attachment well plate (Sigma Aldrich, USA) for subsequent treatments.

### 2.5. Efficacy of FA and Dex in Reducing Lipid Accumulation in Foam Cell Spheroids

The harvested foam cell spheroids were incubated in DM-10 containing either FA or Dex at 0.1 and 1 *μ*g/mL. After 2 days of incubation, the spheroids were embedded in Optimal Cutting Temperature (OCT) compound (VWR, USA) for cryotomy. Using a cryostat (CM3050 S, Leica, Germany), the samples were sectioned into 10-30 *μ*m thick sections and subsequently stained with ORO and nuclear-counterstained with haematoxylin to evaluate FA/Dex efficacy.

### 2.6. Oil Red O Staining

ORO staining was used to detect the presence of lipid vacuoles in the cells. Details of this assay and the following assays are described in the Supplementary Information. Lipid droplets inside the cells were stained red and observed using an inverted optical microscope (IX53, Olympus, Japan). To quantify the number of cells stained with ORO, 25 images at 400X magnification were taken for each well and cell counting was done manually. The percentage of ORO-stained cells per well was calculated. All the samples were done in triplicate. A cell was considered positively ORO-stained, and therefore a foam cell, when intracellular lipid droplets occupied at least 10% of the cytoplasm space, based on visual inspection by an experienced individual.

### 2.7. OxLDL Immunofluorescence Staining

To measure OxLDL uptake, cells were cultured on 15 mm glass cover slips (Chemglass Life Sciences, USA) in 24-well tissue culture plates. After fixation, the cells were stained with Rabbit anti-LDL (Copper oxidized) primary antibody (Abcam, United Kingdom) and Goat anti-Rabbit IgG (H+L) Secondary Antibody, Alexa Fluor® 488 conjugate (Life Technologies, USA), and subsequently mounted with ProLong® Gold Antifade Mountant with 4′,6-diamidino-2-phenylindole (DAPI) (Life Technologies, USA). The images were observed and captured using a confocal laser scanning microscope (Leica TCS SP5, Leica Microsystems, Germany).

### 2.8. PicoGreen Assay

Cell proliferation was evaluated by quantifying double-stranded DNA (dsDNA) in cell lysates using Quant-iT™ PicoGreen® dsDNA kit (Life Technologies, USA). The amount of dsDNA in the sample was quantified against a calibration curve obtained from dsDNA standards provided in the kit.

### 2.9. Cholesterol Assay

CE accumulation in the cells was quantified using Cholesterol/Cholesteryl Ester Quantitation Assay kit (Abcam, United Kingdom). Total and free cholesterol values were calculated against a calibration curve obtained from the supplied cholesterol standards. The amount of CE was determined by subtracting the amount of free cholesterol from the total, and the results were normalized to the dsDNA amount.

### 2.10. Cytokine Array

To detect secreted cytokines, cell culture supernates were collected and examined using a cytokine array (Proteome Profiler™ Human XL Cytokine Array Kit, R&D Systems, USA) according to the manufacturer's instructions. Signal intensities were quantified using the ImageQuant software and normalized to the untreated samples.

### 2.11. Statistical Analysis

All the experiments were performed in triplicates, and quantitative results were expressed as mean ± standard deviation (SD). Data were analysed by One-Way and Two-Way ANOVA with post hoc Tukey's analysis using Origin Pro 2015 (OriginLab, USA). Data presented were representative of 2 or 3 independent experiments. A *p* value below 0.05 was considered statistically significant.

## 3. Results

### 3.1. Foam Cell Formation

**Figure 1:**
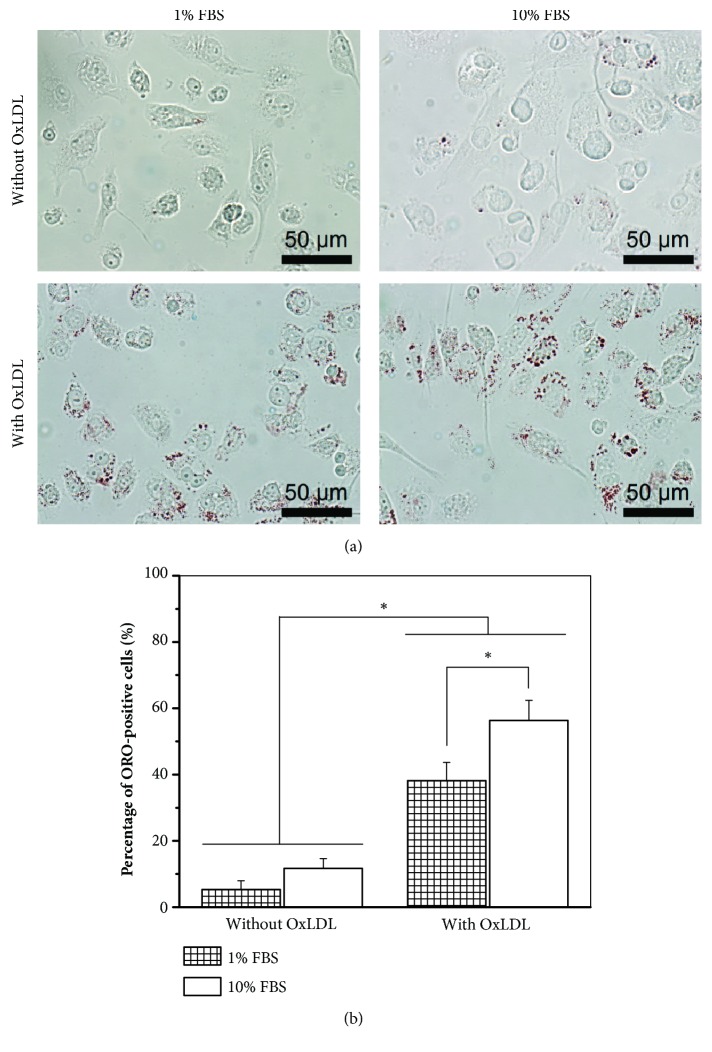
**Characterization of lipid vacuoles in foam cells. **Oil Red O (ORO) staining of THP-1 derived foam cells cultured in media containing either 1% or 10% fetal bovine serum (FBS), and in the absence/presence of 100 *μ*g/mL oxidized low-density lipoproteins (OxLDL). (a) Optical microscopic images of ORO staining. (b) Quantification of foam cell formation based on ORO staining; ^*∗*^ * p* < 0.05. Data presented were representative of 2 independent experiments; n = 3.

**Figure 2:**
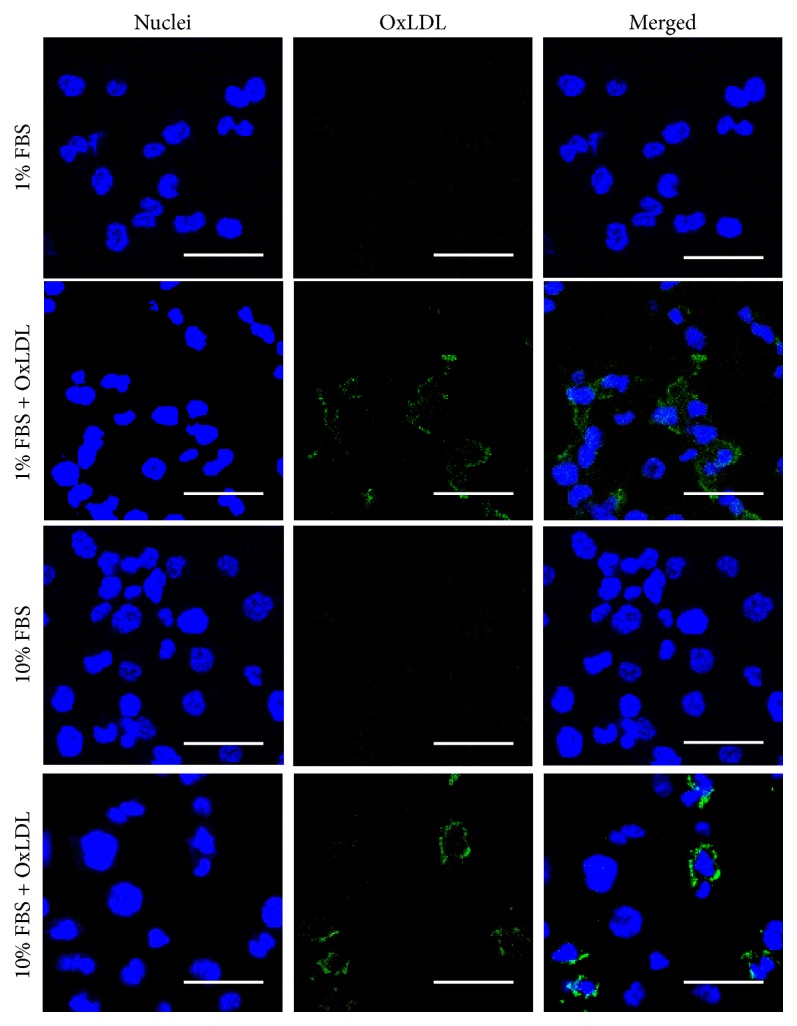
**Examination of OxLDL uptake by foam cells. **Oxidized low-density lipoproteins (OxLDL) immunofluorescence staining of THP-1 derived foam cells cultured in media containing either 1% or 10% fetal bovine serum (FBS) and in the absence/presence of 100 *μ*g/mL OxLDL. Cell nuclei and OxLDL were stained blue and green, respectively. The scale bars represent 50 *μ*m. Images presented were representative of 2 independent experiments; n = 3.

The effects of FBS and OxLDL on foam cell formation were characterized using ORO staining as shown in Figure 1. Even in the absence of OxLDL, 5% and 12% of the total counted macrophages differentiated to foam cells in the presence of 1% and 10% FBS, respectively, in the differentiation medium. The percentages of foam cells increased to 38% and 56%, respectively, when 100 *μ*g/mL OxLDL was fed to the cells. The uptake of OxLDL was then examined by immunostaining as demonstrated in the confocal laser microscopy images in Figure 2. The images illustrated that OxLDL in the medium was uptaken by the foam cells, while no OxLDL was detected in the untreated cells.

### 3.2. Effects of FA and Dex Concentrations on Foam Cell Behaviours

**Figure 3:**
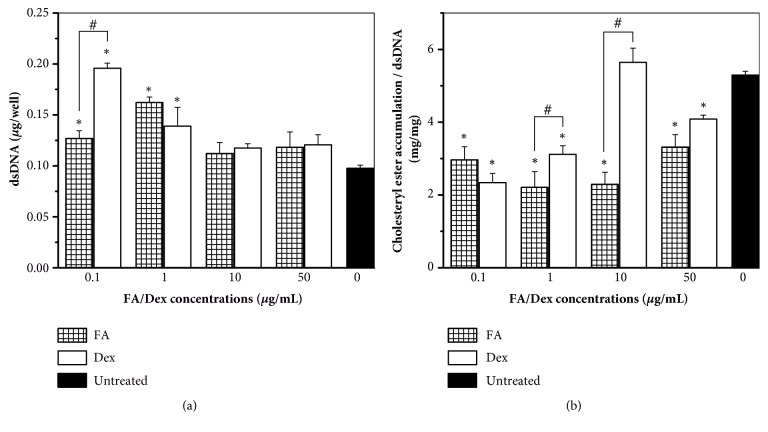
**Effects of FA and Dex on foam cell proliferation and lipid accumulation**. Quantification of double-stranded DNA (dsDNA) and normalized cholesteryl ester accumulation in THP-1 derived foam cells cultured in media containing 10% fetal bovine serum and 100 *μ*g/mL oxidized low-density lipoproteins and treated with different concentrations of Fluocinolone acetonide (FA) and dexamethasone (Dex). (a) dsDNA quantification results obtained from Picogreen assay. (b) Cholesteryl ester accumulation results normalized to the corresponding dsDNA amounts. ^*∗*^ * p* < 0.05 between the indicated groups and untreated controls. ^#^ * p* < 0.05 between FA and Dex at the same concentration. Data presented were representative of 3 independent experiments, n = 3.

Different FA/Dex concentrations, 0.1–50 *μ*g/mL, were used to investigate their influences on the proliferation (Figure 3(a)) and CE accumulation (Figure 3(b)) of foam cells. The results showed that, at lower concentrations of FA/Dex (0.1 and 1 *μ*g/mL), cell numbers were significantly higher while lipid accumulation was significantly lower than untreated cells (*p* < 0.05). The CE amounts in these GC-treated cells were only about half of the untreated cells. At the concentration of 1 *μ*g/mL, although there was no significant difference in cell proliferation between the samples treated with FA and Dex, the cells exposed to FA showed significant reduction in lipid accumulation in comparison with cells exposed to Dex (*p* < 0.05). At higher concentrations of FA/Dex (10 and 50 *μ*g/mL), the number of GC-treated cells was not different from the untreated sample. Nonetheless, the cells treated with FA showed considerably lower CE amounts than untreated cells (*p* < 0.05). Moreover, at 10 *μ*g/mL of FA, lipid accumulation significantly decreased compared to cells treated with Dex at the same concentration (*p* < 0.05).

### 3.3. Cytokine Profile of Foam Cells Treated with FA and Dex

**Figure 4:**
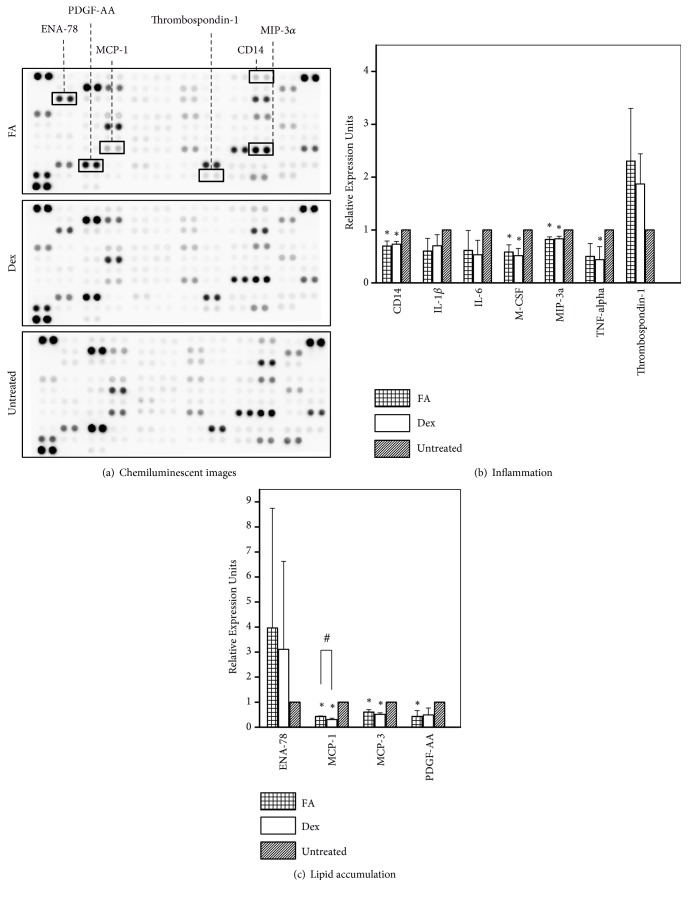
**Evaluation of cytokine release from foam cells treated with FA/Dex**. (a) Chemiluminescent images of cytokine array results from THP-1 derived foam cells cultured in media containing 10% fetal bovine serum and 100 *μ*g/mL oxidized low-density lipoproteins and treated with 1 *μ*g/mL Fluocinolone acetonide (FA)/ dexamethasone (Dex). Cytokines related to inflammation and lipid accumulation that registered differences in secreted levels are labelled in the top panel; (b) and (c) quantification of the cytokine secretions. Cytokines are categorized into two categories: (b) inflammation, and (c) lipid accumulation. ^*∗*^ * p* < 0.05 between the indicated groups and untreated controls. ^#^ * p* < 0.05 between FA and Dex-treated groups. Data presented were representative of 3 independent experiments, n = 3.

Cell culture supernatants of different samples (1 *μ*g/mL FA, 1 *μ*g/mL Dex and untreated) were used to analyse cytokine secretions. The images of cytokine arrays and the quantification data of cytokine secretion related to inflammation and lipid accumulation were presented in Figure 4. There were two groups of cytokines examined in our study: (a)** inflammation**: cluster of differentiation 14 (CD14), IL-1*β*, IL-6, Macrophage colony-stimulating factor (M-CSF), macrophage inflammatory protein-3-alpha (MIP-3*α*, also known as CCL20), TNF-*α*, and Thrombospondin-1 (TSP-1) and (b)** lipid accumulation**: epithelial neutrophil activating peptide-78 (ENA-78, also known as CXCL5), monocyte chemoattractant protein-1 (MCP-1), MCP-3, and platelet-derived growth factor- (PDGF-) AA. For the inflammation-related cytokines, our results showed that the levels of CD14, M-CSF, MIP-3*α*, and TNF-*α* released from the cells treated with FA/Dex were significantly lower than untreated cells (*p* < 0.05, Figure 4(b)). Although FA/Dex downregulated the release of IL-1*β* and IL-6 and upregulated TSP-1 expression compared to the control, these differences were not statistically significant (*p* > 0.05, Figure 4(b)). For the lipid accumulation-related cytokines, our cytokine quantification data indicated that the expression levels of MCP-1, MCP-3, and PDGF-AA were reduced 2–3 times in the cells cultured with the GC compared to non-GC-treated cells (*p* < 0.05, Figure 4(c)). Additionally, ENA-78 secretion by GC-treated samples was higher than the control, although the differences were not statistically significant (*p* > 0.05). Since the cytokine experiment was repeated three times with triplicate samples in each run, we believed that the statistical nonsignificance was mainly contributed by the lack of a significant physiological effect. Comparable levels between FA- and Dex-treated cells were found for MCP-1, MCP-3, PDGF-AA, and ENA-78.

### 3.4. Efficacy of FA and Dex in Reducing Lipid Accumulation in 3D Foam Cell Spheroids

**Figure 5:**
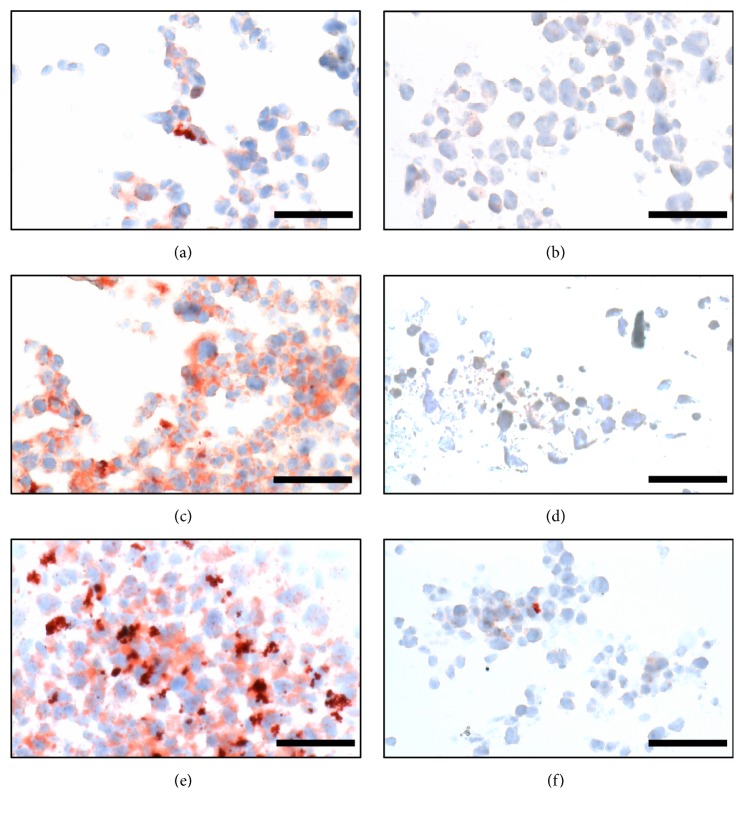
**Characterization of lipid accumulation in 3D foam cell spheroids. **Oil Red O (ORO) and haematoxylin staining of cryosections of foam cell spheroids in different conditions. (a–d) Foam cell spheroids treated with 0.1 *μ*g/mL FA, 1 *μ*g/mL FA, 0.1 *μ*g/mL Dex and 1 *μ*g/mL Dex, respectively; (e) untreated foam cell spheroid; (f) negative control: untreated macrophage spheroid. The scale bars represent 50 *μ*m. Images presented were representative of 3 independent experiments, n = 3.

Foam cell spheroids of 529 ± 56 *μ*m diameters were successfully produced using the hanging droplet culture method, commonly used to induce embryonic stem cell differentiation [20]. The results in Figure 5 showed that, for spheroids treated with 1 *μ*g/mL of FA and Dex, there was a significant reduction of oil droplets as compared to the untreated sample, but there was no significant difference between FA and Dex. At a lower concentration of 0.1 *μ*g/mL, the glucocorticoid efficacy in reducing lipid accumulation in the spheroids was reduced, but, interestingly, FA produced a more significant effect compared to Dex at that concentration.

## 4. Discussion

### 4.1. Foam Cell Formation

LDL uptake by monocyte-derived macrophages is one of the earliest pathogenic events in the nascent plaque. In our study, 5% and 12% of the macrophages were converted to foam cells in DM-01 and DM-10 media, respectively, in the absence of OxLDL (Figure 1(b)), due to the presence of native LDL in the FBS added to the culture media. This LDL could be uptaken by macrophages* via* LDL receptor or pinocytosis, although the concentrations of LDL in the DM-01 and DM-10 media were 0.9 and 9 *μ*g/mL, respectively, which might not be enough to induce pinocytosis. Native LDL could also be oxidized by strong oxidative entities in the medium, such as transition metal ions [21], making it possible to be uptaken* via* scavenger receptors (SR) [1]. However, there was no OxLDL in the cells cultured without supplemented OxLDL (Figure 2), proving that native LDL in our media was not oxidized to OxLDL. Kruth et al. [22] indicated that activation of human monocyte-derived macrophages by PMA-stimulated native LDL uptake by macropinocytosis, which was the likely mechanism of LDL uptake in our setup. Taken together, the formation of foam cells when cultured in media without OxLDL was due to the uptake of native LDL in FBS* via* LDL receptors or PMA-stimulated macropinocytosis.

In the presence of 100 *μ*g/mL OxLDL, the percentages of foam cells significantly increased (Figure 1), due to the ease of OxLDL uptake* via* SR receptors (Figure 2). Although OxLDL was aggressively taken up by macrophages, only up to half of the macrophage population converted to foam cells. This could be due to the fact that OxLDL partially inactivates lysosomal enzymes that degrade LDL, leading to poor metabolism of OxLDL within lysosomes [23], thus limiting the capacity of CE lipid droplet formation. In the absence of OxLDL, the percentage of foam cell formation was independent of FBS concentration; in the presence of OxLDL, 10% FBS produced a significantly higher percentage of foam cells than 1% FBS (Figure 1(b)). Consistent with observations made elsewhere when coincubating native LDL with acetylated LDL [22], coincubation of native LDL with OxLDL potentially increased macropinocytosis mediated uptake of native LDL by macrophages.

Understanding the uptake of LDL and OxLDL by macrophages and subsequent inflammatory response and lipid droplet accumulation in foam cells are essential to explore the capacity of GCs or other drugs to stop or even reverse the process. Figures 1 and 2 therefore helped to confirm that we successfully established foam cell formation through OxLDL uptake by macrophages. The effects of FA/Dex in mitigating the inflammation caused by this OxLDL uptake could therefore be demonstrated in a systematic way in subsequent figures.

### 4.2. The Potential of FA as a Protective Therapeutic for Atherosclerosis

Dex is the most commonly proposed GC for atherosclerotic therapy [10, 12]. In our current work, FA was compared with Dex for its capacity in atherosclerotic treatment. We showed that both FA and Dex helped macrophages survive better (Figure 3(a)) and significantly reduced lipid accumulation compared to untreated cells (Figure 3(b)). In terms of retarding lipid accumulation, FA performed either as well as or better than Dex across the concentrations tested.

In terms of cytokines release, FA and Dex demonstrated tremendous efficacy in suppressing atherosclerotic inflammation compared to the untreated group (Figure 4(b)). High expression levels of proinflammatory cytokines IL-1*β*, IL-6, M-CSF, MIP-3*α*, and TNF-*α* have been known to increase atherosclerotic lesion progression and plaque inflammation [24–26]. Increased levels of CD14 will activate the transcription factor NF-*κ*B and the toll-like receptor (TLR) pathway, when lipopolysaccharides (LPS) binds, to release proinflammatory cytokines into the vasculature [27]. In the current study, proinflammatory cytokines such as CD14, M-CSF, MIP-3*α*, and TNF-*α* were all significantly reduced in the GC-treated cells (Figure 4(b)). In corroboration, Schepers et al. showed that mRNA expression levels of MIP-1*α* and CD14 decreased significantly compared to the control group, after 7 days of Dex treatment in hypercholesterolemic ApoE3-Leiden transgenic mice and led to a significant reduction in vein graft thickening [12]. For all the cytokines in the inflammation category, FA revealed the same trend as Dex, in comparison with the control group. This suggested that FA and Dex acted through similar mechanisms in effecting anti-inflammatory outcomes in THP-1 derived foam cells. OxLDL, which signals* via* the scavenger receptor CD36 together with toll-like receptors TLR4 and TLR6 (CD36-TLR4-TLR6), activates NF-*κ*B signalling pathway and induces inflammatory responses [28]. GCs in the form of GC-GR complexes are able to block the transcriptional activity of NF-*κ*B [7, 8], thereby explaining the anti-inflammatory function of FA and Dex in our study.

FA and Dex also revealed their significant importance in decreasing lipid accumulation compared to the control (Figure 4(c)). MCP-1 is a proinflammatory chemokine, which plays a fundamental role in monocyte recruitment. It also increases the progression of atherosclerosis by promoting lipid oxidation and oxidized lipid accumulation [29]. MCP-3, a chemokine involved in leukocyte trafficking, contributes to the development of atherosclerosis by increasing plasma total cholesterol, atherosclerotic lesions, and hepatic lipid accumulation [30]. The mechanism of PDGF-AA in atherosclerosis is less clear, although its inhibition has been found to reduce lesion formation [31]. In contrast, ENA-78 reveals a protective role in atherosclerosis by increasing expression of the cholesterol efflux regulatory protein ABCA1 and enhancing cholesterol efflux activity in macrophages [32]. In our study, the elevated amounts of secreted ENA-78 along with the reduction of MCP-1, MCP-3, and PDGF-AA resulted in the observed attenuation of lipid accumulation in 2D culture (Figure 3(b)) and 3D model (Figure 5) of FA/Dex-treated samples compared to the untreated sample. Similarly, 7-day Dex treatment reduced MCP-1 mRNA expression about 3 times in hypercholesterolemic ApoE3-Leiden transgenic mice compared to untreated mice [12]. Chono and Morimoto also showed that Dex treatment inhibited CE accumulation in mouse macrophage-derived foam cells [15]. All the cytokines evaluated in the lipid accumulation category showed the same trend between FA and Dex-treated groups in our results.

**Figure 6:**
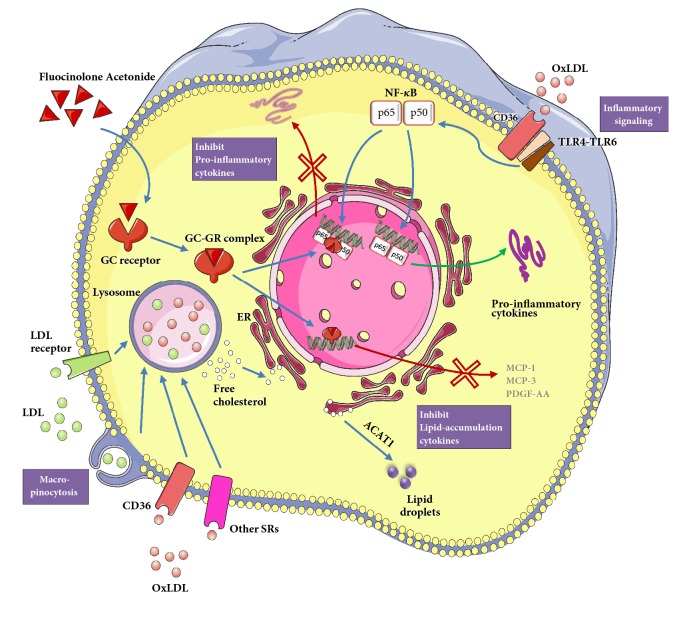
**Proposed mechanisms of lipoprotein uptake and Fluocinolone acetonide effects on inflammation and lipid accumulation in THP-1 derived foam cells**. Native low-density lipoprotein (LDL) is uptaken* via* LDL receptor or macropinocytosis, while oxidized LDL (OxLDL) is uptaken* via* CD36 and other scavenger receptors (SRs). After internalization in the endolysosomal compartment, the cholesteryl esters of native and modified LDL are hydrolysed to free cholesterol and fatty acids. Free cholesterol is then trafficked to the endoplasmic reticulum (ER), where it is reesterified by acetyl-coenzyme A acetyltransferase 1 (ACAT1) to form cholesteryl fatty acid esters. OxLDL, which signals* via* the scavenger receptor CD36 together with toll-like receptors TLR4 and TLR6 (CD36-TLR4-TLR6), activates nuclear factor kappa B (NF-*κ*B) signalling pathway to produce proinflammatory cytokines. Fluocinolone acetonide, a glucocorticoid (GC), can easily diffuse through the cell membrane and bind to glucocorticoid receptors (GRs) in the cytoplasm. GC-GR complexes are rapidly translocated into cellular nuclei and physically interact with NF-*κ*B in the nucleus to block its transcriptional activity. The GC-GR complexes also bind as homodimers to glucocorticoid response elements (GREs), specific DNA sequences in the promoter region of corticosteroid-responsive genes in the nucleus, which leads to a decreased transcription of MCP-1, MCP-3, and PDGF-AA. The reduced levels of these cytokines can help to diminish the lipid accumulation in foam cells.* Modified from original artwork by Servier Medical Art *(https://smart.servier.com/).

The molecular weights of FA and Dex are 452.49 and 392.46 g/mol, respectively. The distribution coefficient, log D, is a measure of the lipophilicity of a drug. It is defined as the ratio between a drug concentration in the organic phase to its concentration in the aqueous phase, at equilibrium of the two immiscible phases, and at an appropriate pH. This value is thus an indirect indication of the solubility of the drug in the membrane and also an indication of the drug's permeability through lipid bilayers. The log D values of FA and Dex at pH 7.4 are 2.56 and 1.95, respectively [33]. These drugs cross cellular membranes by passive diffusion at rates that are directly proportional to their solubility in membranes [34]. Molecular weight is a less important factor in the passive diffusion of drugs. Therefore, FA could diffuse faster than Dex across the plasma membrane, which could explain why FA was superior to Dex in inhibiting lipid accumulation at the therapeutic concentrations of 1 and 10 *μ*g/mL in 2D culture (Figure 3(b)) and at the concentration of 0.1 *μ*g/mL in 3D spheroid model (Figures 5(a) and 5(c)). FA and Dex did not directly interact with LDL/OxLDL to prevent them from being uptaken by the foam cells. They acted* via* regulating cytokines such as MCP-1, MCP-3, and PDGF-AA to prevent lipid accumulation. Based on our findings, we propose the mechanisms of LDL uptake and FA actions in modulating the inflammatory and lipid accumulation response of THP-1 derived foam cells, as shown in Figure 6.

Although many advantages of GCs have been suggested for atherosclerotic treatment, systemic GC treatment has not been adopted clinically because of side effects such as central obesity, insulin resistance, hyperglycaemia, dyslipidaemia, and hypertension [35]. In contrast, local stent-mediated GC delivery was proven to successfully diminish both vascular macrophage infiltration and in-stent neointimal hyperplasia in a porcine coronary stent model [36]. With our current findings, we propose that localized delivery of FA is explored, such as through the development of an injectable FA-loaded nanocarrier system that can target early stages of atherosclerosis, to minimize systemic adverse effects and improve local anti-inflammatory efficacy.

## 5. Conclusions

Foam cell accumulation is a key event in the early stages of atherosclerotic plaque development. The formation of THP-1 derived foam cells was studied in the absence or presence of OxLDL. Without OxLDL, native LDL present in serum-supplemented culture media could induce a small amount of foam cell formation. With 100 *μ*g/mL OxLDL, the percentage of foam cell formation significantly increased due to the ease of OxLDL uptake* via* SR. Fluocinolone acetonide, a glucocorticoid, was explored for the first time as a protective therapeutic in atherosclerosis, in comparison with dexamethasone, the most common GC proposed for atherosclerotic treatment. At 1 *μ*g/mL, FA and Dex considerably improved macrophage survival and reduced lipid accumulation compared to untreated cells. Moreover, FA/Dex substantially reduced the secretion of inflammatory cytokines including CD14, M-CSF, MIP-3*α*, and TNF-*α*. Importantly, FA was shown to be superior to Dex in reducing lipid accumulation as indicated by cholesterol assay and Oil Red O staining results in 2D and 3D cell culture models. In conclusion, this study sheds light on the potential of using fluocinolone acetonide as an alternative glucocorticoid for modulating foam cell behaviour.

## Data Availability

The data used to support the findings of this study are available from the corresponding author upon request.
