# Supplementary material for: The Potential of Fluocinolone Acetonide to Mitigate Inflammation and Lipid Accumulation in 2D and 3D Foam Cell Cultures
Source: Biomed Res Int. 2018 Nov 22;2018:3739251. doi: 10.1155/2018/3739251 (PMC6282138; doi:10.1155/2018/3739251)
Supplement: Supplementary Materials — are provided in .docx file. [file 3739251.f1.docx]

*Manuscript title*: **The Potential of Fluocinolone Acetonide to Mitigate Inflammation and Lipid Accumulation in 2D and 3D Foam Cell Cultures**

*Authors*: Luong T. H. Nguyen, Aristo Muktabar, Jinkai Tang, Yee Shan Wong, C Shad Thaxton, Subbu S Venkatraman, and Kee Woei Ng

**SUPPLEMENTARY INFORMATION**

**MATERIALS AND METHODS**

## *Oil Red O staining*

ORO (Sigma Aldrich, USA) stock solution of 0.4% w/v was prepared in isopropanol (IPA, Fisher Chemical, USA). ORO working solution was subsequently made up fresh for each time use by diluting the stock solution in deionized (DI) water at the volume ratio of 3:2 and filtering through a 0.2 μm syringe filter (Minisart® high flow syringe filter, Sartorius AG, Germany). Cells were rinsed twice with ice-cold phosphate-buffered saline (PBS, Pan Biotech, Germany) and then fixed in 4% ice-cold formaldehyde (Sigma Aldrich, USA) for 30 min at room temperature. After washing twice with DI water, the cells were soaked in 60% IPA for 5 min and then stained with the ORO working solution for 15 min at room temperature. The cells were subsequently rinsed twice with DI water and twice with 60% IPA before mounted with glass cover slips using an aqueous slide mounting medium (Glycerol gelatin, Sigma Aldrich, USA).

## *OxLDL immunofluorescence staining*

After two washes with ice-cold PBS, the cells were fixed in 4% ice-cold formaldehyde for 1 hr at room temperature. Subsequently, the cells were rinsed three times with PBS containing 0.05% TWEEN 20 (Sigma Aldrich, USA; PBST). A blocking solution of 1% bovine serum albumin (BSA, Sigma Aldrich, USA) in PBS was then applied for 1 hr at room temperature. Rabbit anti-LDL (Copper oxidized) primary antibody (Abcam, United Kingdom) was diluted 100-fold in the blocking solution and consequently incubated with the cells overnight at 4^o^C. The next day, the cells were rinsed three times with PBST for 5 min each and then incubated with a secondary antibody (Goat anti-Rabbit IgG (H+L) Secondary Antibody – Alexa Fluor® 488 conjugate, Life Technologies, USA) at a dilution of 1:100 in the blocking solution, for 1 hr at room temperature and in the dark. After washing three times with PBST for 5 min each, the cover slips were taken out and mounted onto microscope slides using ProLong® Gold Antifade Mountant with 4',6-diamidino-2-phenylindole (DAPI) (Life Technologies, USA).

***PicoGreen assay***

Firstly, the cells were rinsed twice with ice-cold PBS and lysed using a cell lysis buffer (Cat. 9803S, Cell Signaling Technology, USA) on ice for 5 min. Subsequently, two freeze-thaw cycles were performed to further disrupt the cells through ice crystal formation. Each cycle included freezing for 10 min at -80^o^C and complete thawing at room temperature. After these cycles, 100 μL of each sample was transferred to a black 96-well plate (Bibby Sterilin, United Kingdom) and mixed with 100 μL of PicoGreen working solution. The mixture was consequently incubated for 5 min at room temperature in the dark, and the fluorescence intensity was measured using a microplate reader (Infinite M200, Tecan, Switzerland) with an excitation wavelength of 480 nm and an emission wavelength of 520 nm.

***Cholesterol assay***

After two washes with ice-cold PBS, cellular lipids were extracted in a mixture of hexane-IPA (3:2) for 1 hr at room temperature on a shaker (The Belly Button^®^ Shaker, Denville Scientific, USA). The liquid (organic phase) was then transferred to a new plate and air-dried to completely remove the organic solvents. The dried lipids were subsequently dissolved in the cholesterol reaction buffer with 0.05% NP-40 (Tergitol^®^ solution, Sigma Aldrich, USA), and 50 μL of each sample was pipetted to a black 96-well plate. 50 μL of either the total cholesterol reaction mix (with cholesterol esterase) or the free cholesterol reaction mix (without cholesterol esterase) was then added to each well. Consequently, the mixtures were incubated for 1 hr at 37^o^C in the dark. The fluorescence intensity was then measured using the microplate reader with an excitation wavelength of 535 nm and an emission wavelength of 587 nm.

***Cytokine array***

Briefly, the supplied blocking buffer was first applied to the membrane for 1 hr at room temperature. 500 μL of the supernate was then diluted to a final volume of 1.5 mL with the blocking buffer and incubated with the membrane overnight at 4^o^C on a shaker. The next day, the sample was rinsed three times for 10 min each with the wash buffer and subsequently incubated with the detection antibody cocktail for 1 hr at room temperature on a shaker. After three washes with the wash buffer, the array was incubated with Streptavidin-HRP for 30 min at room temperature on a shaker. The membrane was again washed three times and incubated with 1 mL of the chemiluminescence development mix for 1 min at room temperature in a plastic sheet protector. The array signals were consequently captured using a luminescence image analyser (ImageQuant™ LAS 4000 mini, GE Healthcare Life Sciences, USA).
